# Mitral and Tricuspid Transcatheter Interventions Current Indications and Future Directions

**DOI:** 10.3389/fcvm.2020.00061

**Published:** 2020-05-15

**Authors:** Mirjam Gauri Winkel, Fabien Praz, Peter Wenaweser

**Affiliations:** ^1^Department of Cardiology, Inselspital, Bern University Hospital, University of Bern, Bern, Switzerland; ^2^Heart Clinic Hirslanden Zurich, Zurich, Switzerland

**Keywords:** valvular heart disease, mitral interventions, tricuspid interventions, cardioband, mitraclip, pascal, annuloplasty, edge-to-edge repair

## Abstract

Valvular heart disease is responsible for a high rate of morbidity and mortality, especially in the elderly population. With the emergence of new transcatheter treatment options, the therapeutic spectrum for patients with valvular heart disease has considerably expanded during the past years. Interventional treatment of the mitral and tricuspid valve requires an individualized and versatile approach owing to the different etiologies of valvular dysfunction and the complex anatomy of the atrioventricular valves. This article aims to review recent developments, summarize the evidence, indications and limitations of the available systems, and provide a glimpse into the future of transcatheter interventions for the treatment of mitral and tricuspid valve disease.

## Introduction

Valvular heart disease is a common condition that has long been underappreciated although it has tremendous impact on mortality and morbidity. Population-based analyses have shown a prevalence of moderate to severe mitral and tricuspid valve disease of 9.3 and 4.0% in the elderly population, respectively (1, 2). The incremental annual costs for valvular heart disease in the US are substantial [estimated at $23.4 billion (3)] and expected to further increase given population growing and aging.

In the past, a high proportion of patients with severe mitral or tricuspid valve disease has been denied treatment beyond medical therapy, mainly because of age and decreased left ventricular function, both associated with high surgical risk (4). Over the last years, the interventional landscape has widely expanded with new transcatheter methods emerging for both mitral and tricuspid valve treatment. In contrast to transcatheter aortic valve implantation, the interventional treatment of mitral and tricuspid valve disease requires a more versatile approach due to the different etiologies of valvular dysfunction. This article aims to review the evolution of the field, to discuss current indications and limitations and attempts to provide a glimpse to the future of transcatheter interventions for mitral and tricuspid valve disease.

## Transcatheter Mitral Valve Interventions

### State of the Art

The treatment of the mitral valve has long been exclusively based on surgical mitral valve repair or replacement. However, open-heart surgery has several limitations including low penetrance and increased mortality in elderly patients and those with diminished left ventricular function (5). To further improve patient care and expand the therapeutic options of severe mitral valve disease, minimal-invasive percutaneous solutions have been introduced into clinical practice. Current transcatheter interventions are mostly derived from surgical procedures. Based on their mode of action, they can be classified into four groups: leaflet approximation, direct and indirect annuloplasty, chordal, and valve replacement.

The MitraClip® (Abbott Vascular, Chicago, US) device is the first transcatheter technology with CE mark and FDA approval for the treatment of both primary and secondary MR (6). Since its first implantation in 2003, over 100,000 procedures have been performed worldwide. Owing to its minimal-invasive approach and limited interaction with the native anatomy, it carries a high safety profile and enables fast patient's recovery (7, 8).

The first randomized trial to assess the efficacy of the MitraClip system was the EVEREST II trial. Two hundred and seventy nine patients (27% with secondary MR) were assigned to either transcatheter or surgical treatment. At 5 years, there was no difference in terms of mortality between groups (20.8 vs. 26.8%; *p* = 0.4). In primary MR, the primary endpoint (freedom from death, MV surgery or reoperation, and 3+ or 4+ MR) occured more frequently in the transcatheter group (54.5 vs. 23.8%; *p* < 0.001), mainly driven by a higher rate of surgery for recurrent MR, whereas there was no significant difference in patients with secondary MR (59.5 vs. 71.4%; *p* = 0.43). A landmark analysis beyond 6 months through 5 years showed comparable long-term durability with a rate of freedom from surgery for mitral valve dysfunction of 77.7% with MitraClip vs. 76.2% with surgery (*P* = 0.77) (9).

In 2018, two randomized prospective trials comparing optimal medical treatment (OMT) alone to MitraClip in addition to OMT for patients with severe MR and heart failure have been presented. Despite high procedural success in the MitraClip group (92% with MR ≤ 2+), the French MITRA-FR study failed to show any difference for the primary composite endpoint of all-cause mortality and hospitalization for heart failure throughout 2 years (67.1% for OMT vs. 63.8% for MitraClip, HR 1.01) (10). In contrast, the US-American COAPT study showed a relative risk reduction of 47% (number needed to treat of 3.1) of the primary endpoint of rehospitalization for heart failure, as well as 37% of the secondary composite endpoint of rehospitalization or all-cause mortality after 2 years of follow-up. Procedural success was high (98%), durable throughout 2 years (95%), and the procedure was safe (no peri-procedural complications in 97% of patients) (11). Recently, the 3-year follow up data have been presented, reinforcing the previous results with an even larger symptomatic and survival benefit in the interventional group as compared to the OMT arm (12). Table 1 highlights the most important differences between the two studies.

**Table 1:** Key differences between the MITRA-FR and COAPT study [adapted from Praz et al. (13)].

	**MITRA-FR**	**COAPT**
Patients (screened)	304 (452)	614 (1,576)
Age (years)	70 ± 10	72 ± 12
Key exclusion criteria	NYHA class < II	NYHA class < II Advanced heart failure (ACC/AHA Stage D)
	CABG or PCI within 30 days	Untreated CAD requiring revascularization or CABG/PCI within 30 days
	–	Right-sided CHF with advanced right ventricular dysfunction
	–	COPD with home oxygen therapy or chronic oral steroid use
	–	Severe tricuspid regurgitation
	–	sPAP > 70 mmHg unresponsive to vasodilator therapy
Mean LVEF (%)	33 ± 7	31 ± 10
MR EROA (cm^2^)	0.31 ± 0.1	0.41 ± 0.15
LVEDVi (ml/m^2^)	135 ± 35	101 ± 34
Complications (%)^*^	14.6	8.5
Death or hospitalization for HF at 1 year (%)	54.6	33.9
OMT	51.3	46.5
Death or hospitalization for HF at 2 years (%)	63.8	45.7
OMT	67.1	67.9

CAD, coronary artery disease; PCI, percutaneous coronary intervention; CABG, coronary artery bypass grafting; CHF, congestive heart failure; COPD, chronic obstructive pulmonary disease; LVEF, left ventricular ejection fraction; EROA, effective regurgitant orifice area; LVEDVi, indexed left ventricular end-diastolic volume; PHT, pulmonary hypertension; PAP, pulmonary artery pressure.

* *According to MITRA-FR definition*.

The reasons for the different outcomes of these at first sight similar studies are the topic of an ongoing debate between experts coming from the interventional and heart failure community. When interpreting the above-mentioned results, the following factors need to be considered:

1. Discrepancies in the definition of MR severity according to the European or American guidelines (14, 15),
2. Exclusion of patients with severe left ventricular dilation as well as end stage heart failure in COAPT,
3. Supervision of OMT implementation by a central multidisciplinary committee in COAPT,
4. Partly missing echocardiographic and clinical follow-up data in MITRA-FR.

Compared to COAPT, patients in MITRA-FR had a higher rate of peri-procedural complications (14.6 vs. 8.5%) as well as a lower rate of sustained MR reduction (MR ≤ 2+ after 1 year in 83% of the patients vs. 95% in COAPT). This is noteworthy, since substudies of COAPT have shown residual MR (grade 3+/4+) at 30-days to be a predictor of mortality at 2-years of follow-up.

While the state of knowledge from clinical trials and experience is constantly evolving, the MitraClip device itself has experienced only minor modifications, the most important being the introduction of the XTR system with extended clip arms in 2018 (16).

**Figure 1:**
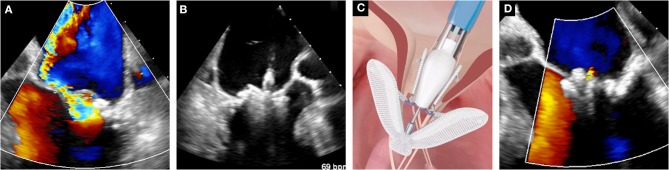
Case of leaflet approximation with PASCAL device in secondary MR. **(A)** Baseline color-Doppler echocardiographic assessment of MR. **(B)** Leaflet clasping. **(C)** Schematic depiction of the PASCAL system (courtesy of Edwards Lifesciences). **(D)** Assessment of the final result with color Doppler echocardiography.

The PASCAL transcatheter mitral valve repair system (Edwards Lifesciences, Irvine, USA) has been developed to overcome some of the intrinsic technical limitations of the hitherto existing system. It has larger and longer arms, a central spacer designed to fill the coaptation defect and decrease the tension applied on the valve tissue, as well the capability to grasp each leaflet separately. After a preliminary encouraging compassionate experience in 23 patients (17), feasibility and efficacy in reducing MR have been recently confirmed in a multicenter prospective trial (18). Subsequently, the PASCAL system has gained CE mark in February 2019 for the treatment of both primary and secondary MR. Figure 1 shows an illustrative case of severe secondary MR successfully treated with implantation of one PASCAL implant.

Direct annuloplasty devices, anchored into the mitral valve annulus, and indirect annuloplasty devices aim to reduce annular dimensions and hence, increase leaflet coaptation in secondary MR. The Carillon Mitral Contour System® (Cardiac Dimensions, Kirkland US) was the first indirect annuloplasty device to receive CE mark (19). The recently published REDUCE FMR study was a randomized sham-controlled trial including 120 patients, which showed a statistically significant reduction in mitral regurgitant volume as well as left ventricular volumes in the interventional group compared to OMT alone (20). Over 800 procedures have been performed so far. Other indirect annuloplasty devices are under clinical evaluation, like the ARTO® device (MVRX, San Mateo US) (21) and the Mitral Loop Cerclage Catheter System® (Tau-PNU Medical, Seoul KOR) (22), but have not received commercial approval yet. The only direct annuloplasty device commercially available is the Cardioband® system (Edwards Lifesciences, Irvine US), with favorable early results in a study of 60 patients (23). At 12 months, seven patients (12%) required a second intervention due to recurrent MR and 61% had moderate or less MR (24).

Transcatheter chordal repair is an off-pump surgical procedure through a transapical access aiming to restore physiological leaflet movement through the implantation of artificial ePTFE chords. The Harpoon® (Edwards Lifesciences, Irvine US) and the NeoChord® (NeoChord, St. Louis Park US) systems are based on this principle and are both commercially available. Harpoon has received CE mark after the recent presentation of the 1-year outcome data of the Mitral TRans-Apical neoChordal Echo-guided Repair (TRACER) Trial. Neochord has the largest experience with 1,200 patients treated so far. Safety and feasibility as well as durability could be demonstrated in the TACT trial and by registry data (25–27).

While transcatheter treatment options for mitral valve repair have evolved rapidly over the last years, the field of transcatheter mitral valve replacement (TMVR) is still in its infancy, mainly limited by the high rate of screening failure due to unsuitable anatomy. Advantages and disadvantages of TMVR compared to repair techniques are summarized in Table 2. Two approaches are currently being pursued: the off-label use of transcatheter heart valves that have been originally designed for the aortic position (mainly for valve-in-valve, valve-in-ring and valve-in-MAC interventions) and the use of dedicated devices. The recently reported early results of the global feasibility trial with the Tendyne® prosthesis (Abbott Structural, Santa Clara US) represent the largest experience with TMVR in native mitral valves. The system has obtained CE mark approval in January 2020. Despite high technical success and procedural safety, all-cause mortality and hospitalization for heart failure at 1-year follow-up were both substantial (26 and 31%, respectively) (28, 29). Several other devices are currently evaluated in clinical trials, such as the Intrepid® TMVR system (Medtronic, Minneapolis US) for which favorable results have been reported in a pivotal study (30). Among the issues that still need to be addressed, obstruction of the left ventricular outflow tract, valve thrombogenicity and access route are probably the most salient.

**Table 2:** Advantages and disadvantages of transcatheter mitral valve repair and replacement.

**Transcatheter mitral valve repair**	**Transcatheter mitral valve replacement**
High procedural safety	Some procedural risks
MR reduction anatomy-dependent and not always predictable	High efficacy in terms of MR reduction (one system fits all pathologies?)
Limited interaction with the native anatomy	Risk of LVOT obstruction and interaction with the subvalvular apparatus
Low thrombogenicity	Elevated risk of valve thrombosis
Risk of MR recurrence during long-term (?)	Durable result (?)

### Current Indications and Limitations

In the field of mitral valve disease, surgical treatment continues to play a predominant role. Nonetheless, transcatheter treatment is gaining increased significance for specific clinical scenarios and poses a viable treatment option for well-selected patients.

#### Primary MR

Surgery represents the standard of care for primary MR owing to excellent efficacy and long-term results of mitral valve repair and should be preferred over replacement when valve anatomy is suitable and perioperative risk acceptable (15, 31). In patients with prohibitive surgical risk (generally due to age), a transcatheter treatment should be evaluated by the interdisciplinary Heart Team. Depending on the individual anatomy of the valve and the subvalvular apparatus, an edge-to-edge repair, chordal replacement or mitral valve replacement can be considered (32).

#### Secondary MR

Guideline-directed medical treatment together with cardiac resynchronization (if indicated) are the essential initial therapeutic steps. In patients with persisting symptomatic MR despite these measures, a corrective intervention should be considered. Evidence supporting surgical treatment in this setting is weak and survival benefit as well as result durability are uncertain (33, 34). However, patients who require coronary artery bypass grafting should undergo concomitant surgical valve treatment of severe MR (15). The eligibility for edge-to-edge repair, annuloplasty or valve replacement has to be assessed individually using multimodality imaging. The combination of different devices, e.g. different edge-to-edge systems or edge-to-edge and annuloplasty, may offer an even more individualized approach.

#### Bridge to Circulatory Support or Heart Transplantation

Patients with advanced heart failure should be first evaluated for circulatory support and heart transplantation. As an adjunctive option, edge-to-edge reconstruction of the mitral valve may stabilize the disease and hence, delay the need for a left-ventricular assist device. In COAPT, progression of disease in terms of further decrease in left ventricular function and increase in LV dilation could be contained using the MitraClip (35) and the number of implanted assist-devices and heart transplants was significantly lower in the interventional group (4.4 vs. 9.5%, *P* = 0.01) (11).

#### Acute Severe Mitral Regurgitation

Acute mitral regurgitation usually requires prompt surgical treatment while time to surgery can be bridged by stabilization with an intra-aortic balloon pump (IABP) or Impella in addition to medical therapy (36). Several case reports and registry data have reported emergent edge-to-edge reconstruction to be a feasible alternative in selected patients (37–40).

#### Rheumatic Mitral Stenosis

Given favorable valve morphology, percutaneous balloon valvuloplasty remains the treatment of choice in symptomatic patients (15). In heavily calcified anatomies, concomitant mitral regurgitation or severe subvalvular disease, surgical intervention is generally the only option.

#### Valve-in-Valve, Valve-in Ring and Valve-in-MAC

Valve-in-valve treatment of degenerated bioprostheses in the mitral position is an option, especially in older patients with prohibitive risk for a redo surgical procedure. A recently published multicenter registry has shown excellent results for ViV procedures, whereas Valve-in-ring and TMVR in heavy calcified mitral valves (MAC) were associated with rather high rates of procedural complications and mortality (41). The use of a dedicated prosthesis in patients with MAC may lead to better clinical outcomes as demonstrated by the recent experience with the Tendyne valve (42). For mitral valve replacement strategies, an important limitation is the potential obstruction of the left ventricular outflow tract (LVOT) that needs to be evaluated carefully by pre-procedural imaging like 4D computed tomography and 3D-reconstruction, as well as 3D-printing for implantation simulation (43–45). Intentional laceration of the anterior mitral valve leaflet (LAMPOON) or preemptive alcohol septal ablation have been introduced as techniques to prevent outflow obstruction (46, 47).

### What the Future Holds

Further research is needed to broaden the evidence of transcatheter devices, observe long-term clinical outcomes and durability, and evaluate procedural success in larger patient cohorts. Several ongoing studies aim to show the benefit of transcatheter treatment compared to medical therapy, surgical treatment, or in head-to-head comparison of different devices. The CLASP IID/F study, for example, evaluates the safety and efficacy of the PASCAL system compared to the MitraClip system in a non-inferiority design. Other transcatheter devices are currently awaiting commercial approval by the FDA and/or CE mark, like the Millipede IRIS® (Millipede, Boston Scientific, Marlborough US) for direct annuloplasty (48). New iterations of the MitraClip system will provide wider clip arms, direct measurement of left atrial pressure, as well as optional independent leaflet grasping. The RESHAPE-HF2 trial (RandomizEd Study of tHe MitrACliP DEvice in Heart Failure Patients With Clinically Significant Functional Mitral Regurgitation) aims to provide further evidence for the use of the MitraClip System in chronic heart failure patients (49), whereas the MATTERHORN trial (Mitral vAlve reconsTrucTion for advancEd Insufficiency of Functional or iscHemic ORigiN) is comparing MitraClip to reconstructive mitral valve surgery (50). Research seeking better understanding of the pathophysiology and natural history of disease progression will enable better patient selection and targeted, individualized device therapy.

Incipiently, many devices have been developed for the transapical access, but especially in patients with secondary MR and preexisting LV dysfunction, this approach has been associated with high mortality. Currently, existing systems are modified and miniaturized to be introduced via the transfemoral/-septal access, thereby reducing the invasiveness of the procedure and decreasing peri-procedural complications. Compared to mitral valve repair, valve replacement has the advantage that, especially in patients with a complex anatomy, a nearly complete resolution of MR can be achieved. In addition, the subvalvular apparatus remains intact. The future of transcatheter mitral valve interventions might therefore be a transseptal valve replacement in the vast majority of patients, but several technical challenges have to be overcome and open questions, like the optimal patient selection and appropriate antithrombotic management, need to be addressed upfront.

## Tricuspid Valve Interventions

### State of the Art

Severe tricuspid regurgitation (TR) has been neglected in the past, as it is mainly associated with left-sided heart problems, but also often underestimated due to challenging imaging and grading. Meanwhile, several studies have shown TR to be an independent prognostic predictor of worse clinical outcome and poor survival (2, 51, 52). However, due to age, co-morbidities and generally advanced stage of the disease, surgical treatment is often no longer a reasonable option at the time of clinical presentation. Nevertheless, the case volume of tricuspid valve repair and replacement both have increased over the last years, but in-hospital mortality after tricuspid valve surgery remains stable (8.8%) (53, 54). In addition, surgery is associated with a longer hospital stay and substantial costs.

Subsequently, new transcatheter treatment options have emerged. Since most procedures were adapted from left atrioventricular valve interventions, they follow similar underlying principles: edge-to-edge repair enhancing leaflet coaptation, annuloplasty aiming for annular size reduction and valve replacement.

The most widely used transcatheter technique so far is the MitraClip system with encouraging results (55–58): reverse remodeling of the right ventricle (59) and improved cardiac output (60). In patients with previously elevated liver enzymes due to congestion a significant reduction could be demonstrated (61).

The Cardioband direct annuloplasty system (Edwards Lifesciences, Irvine US) has recently obtained CE approval for the treatment of functional TR, based on the results of a prospective observational study, which showed high technical success (100%) and significant improvement of functional status (88% of patients in NYHA class I-II after 6 months) (62).

Other transcatheter approaches, such as direct annuloplasty using a caval anchoring stent, the TriCinch system, 4TECH, Galway IRL (63) or the implantation of bicaval stenting devices [e.g., TRICENTO, NVT, Hechingen DE (64)], are currently under clinical investigation.

Transcatheter tricuspid valve replacement (65) is a promising alternative. However, complete resolution of TR may be critical in patients with advanced RV dysfunction due to potential RV failure especially in patients with previously described pulmonary hypertension (66).

The hitherto evidence is largely derived from registry data and case reports. The TriValve Registry is the first large scale international database collecting data on transcatheter tricuspid valve interventions. The mid-term results reported a procedural success rate of 72.8% with no difference among the different devices (66% MitraClip, 9% CAVI, 8% FORMA, 6% Trialign, 4% Cardioband, 4% TriCinch, 3% others). Procedural failure (residual TR ≥ grade 2+ was identified as a predictor of adverse outcomes. Peri-procedural mortality was 0% and MACCE occurred in 10.3% at 30-days follow-up (67).

A recently published propensity matched case-control study comparing transcatheter valve therapy to medical treatment alone further support the current development. In the 268 patients from the TriValve registry that were matched to solely medically managed patients, TTVI was associated with a survival benefit (mortality 23 ± 3% vs. 36 ± 3%, *p* = 0.001), as well as a reduction of rehospitalization for heart failure (26 ± 3% vs. 47 ± 3% *p* < 0.0001) at 1-year follow-up (68).

### Current Indications And Limitations

Transcatheter tricuspid valve interventions (TTVI) pose a number of anatomical and technical challenges. Thinner leaflets and larger coaptation gaps render leaflet approximation more difficult than on the left side. Routine use of the PASCAL and the new iteration of the MitraClip XTR system, both featuring extended arms, may facilitate grasping (69).

**Figure 2:**
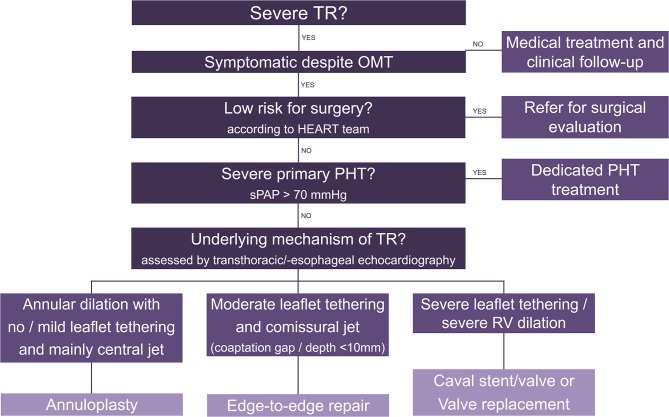
Decision algorithm for transcatheter tricuspid valve interventions. TR, tricuspid regurgitation; OMT, optimal medical therapy; PHT, pulmonary hypertension; RV, right ventricle.

The choice of the right treatment option for patients with severe TR remains the main challenge. Figure 2 attempts to propose a possible decision algorithm based on clinical experience and the data available so far. Importantly, patients with TR induced by a cardiac implantable electronic device lead may also benefit from transcatheter treatment and the same decision criteria apply. To determine eligibility for annuloplasty, caval stent/valve implantation and valve replacement, advanced imaging including multidetector computed tomography and 3D TEE are necessary. In case of advanced disease, combined procedures may be evaluated (e.g., sequential annuloplasty and edge-to-edge repair).

**Figure 3:**
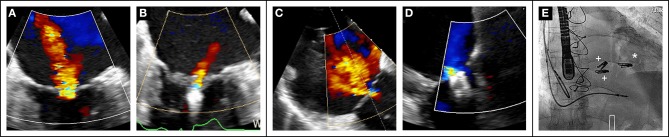
Case of combined mitral and tricuspid edge-to-edge repair in a 79-year old sympatomatic (NYHA III) male patient with prohibitive surgical risk (Euroscore II 21%). Baseline transesophageal assessment showed severe MR **(A)**. After transseptal puncture, the mitral valve was treated first with the placement of one MitraClip XTR. with MR reduction to grade 1+ **(B)**. Severe TR at baseline **(C)** Two MitraClip XTR were placed in the anteroseptal commissure with reduction of TR to mild **(D)**. Final fluoroscopic result ((**E**), *MitraClip mitral, + MitraClips tricuspid).

Recent data (70) also suggest the importance of a global approach to combined mitral and tricuspid valve disease as illustrated in Figure 3.

### What the Future Holds

Patients with severe TR represent a complex and heterogeneous population, and identifying the optimal method and timing of treatment are crucial. Strategies adapted to the individual stage of the disease are necessary. Further research is needed to better understand the incidence, pathophysiology and underlying mechanisms governing disease progression. Development of new and validation of established imaging techniques are required for more accurate and reproducible grading of TR severity, as well as anatomical screening.

Dedicated prospective randomized trials are necessary to determine the true clinical benefit of TR correction.

## Summary

Over the last years, numerous transcatheter techniques for the treatment of mitral and tricuspid valve disease have been introduced. Patient volume will substantially increase over the next years and clinical indications further expand. In contrast to transcatheter aortic valve implantation, percutaneous treatment of the mitral and tricuspid valves will to a lesser extent represent an alternative to surgery, but rather address the needs of a large population of patients that has been undertreated so far.

## Author Contributions

All authors have made substantial contributions to the conception of the work. It has been drafted by MW and FP and has been critically revised by all authors for important intellectual content. All authors have given their approval for publication of the content and have agreed to be accountable for all aspects of the work in ensuring that questions related to the accuracy or integrity of any part of the work are appropriately investigated and resolved.

## Conflict of Interest

PW received lecture and proctoring fees from Edwards Lifesciences and Medtronic. The remaining authors declare that the research was conducted in the absence of any commercial or financial relationships that could be construed as a potential conflict of interest.
